# Update on Vitamin D Status and Seasonal Variation in a Non-Supplemented Population Living in a High Polluted Urban Area—A Cross-Sectional Study

**DOI:** 10.3390/nu18101614

**Published:** 2026-05-20

**Authors:** Francesco Bertoldo, Renata Bortolus, Francesca Filippini, Francesca Chiaffarino, Silvia Udali, Monica Rizzi, Rachele Montemezzi, Giorgio Gandini, Martina Montagnana, Giuseppe Lippi, Sara Moruzzi, Fabio Parazzini, Nicola Martinelli, Matteo Lombini, Sergio De Marchi, Francesca Pizzolo, Simonetta Friso

**Affiliations:** 1 Unit of Internal Medicine D, Department of Medicine, University of Verona, 37124 Verona, Italy; matteo.lombini@univr.it; 2 Office for Research Promotion, Azienda Ospedaliera Universitaria Integrata AOUI Verona, 37126 Verona, Italyfrancesca.filippini83@gmail.com (F.F.); 3 Gynaecology Unit, Fondazione Istituto di Ricovero e Cura a Carattere Scientifico (IRCCS) Ca’ Granda Ospedale Maggiore Policlinico, 20122 Milan, Italy; francesca.chiaffarino@policlinico.mi.it; 4 Unit of Internal Medicine B, “Epigenomics and Gene-Nutrient Interactions” Laboratory, Department of Medicine, University of Verona, 37124 Verona, Italy; silvia.udali@gmail.com (S.U.); sara.moruzzi@aovr.veneto.it (S.M.); nicola.martinelli@univr.it (N.M.); francesca.pizzolo@univr.it (F.P.); simonetta.friso@univr.it (S.F.); 5 Unit of Transfusion Medicine, Azienda Ospedaliera Universitaria Integrata AOUI Verona, 37126 Verona, Italy; monica.rizzi2@aovr.veneto.it (M.R.); rachele.montemezzi@aovr.veneto.it (R.M.); giorgio.gandini@aovr.veneto.it (G.G.); 6 Department of Medicine—DIMED, University of Padua, 35122 Padua, Italy; martina.montagnana@unipd.it; 7 Section of Clinical Biochemistry, Department of Neurosciences, Biomedicine and Movement Sciences, University of Verona, 37124 Verona, Italy; giuseppe.lippi@univr.it; 8 Department of Clinical Sciences and Community Health, Università degli Studi di Milano, 20122 Milan, Italy; fabio.parazzini@unimi.it; 9 Unit of Angiology, Department of Medicine, University of Verona, 37124 Verona, Italy; sergio.demarchi@univr.it

**Keywords:** vitamin D, serum sample, seasonality, air pollution, Italy

## Abstract

**Background:** Serum concentrations of 25-hydroxyvitamin D [25(OH)D] are associated with the risk of several chronic and acute diseases. However, updated data on vitamin D status in Mediterranean countries, including Italy, remain limited, hindering effective public health strategies. **Objective:** To assess serum 25(OH)D levels and their seasonal variation in healthy blood donors aged 18–65 years living in Northern Italy and not taking vitamin D supplements. Given the latitude and the high levels of environmental pollution, cutaneous vitamin D synthesis may be impaired in this population. Recent Italian guidelines on supplementation emphasize the need for updated data on the prevalence of hypovitaminosis D and seasonal variation in endogenous vitamin D synthesis. **Methods:** In this exploratory retrospective cross-sectional study, 534 blood donors (268 men and 266 women) attending the Transfusion Medicine Unit of the Verona University Hospital were enrolled between April 2016 and May 2018. Serum 25(OH)D concentrations were analyzed by season. Clinical, lifestyle, pharmacological and dietary characteristics were also collected. **Results:** Among healthy, normal-weight individuals, the prevalence of vitamin D insufficiency (25(OH)D < 50 nmol/L) was low and limited to one-two months per year. Overweight and obesity significantly reduced the likelihood of achieving adequate 25(OH)D levels through cutaneous synthesis for several months. Mean 25(OH)D concentrations were higher than those previously reported in the same area, while seasonal variation remained preserved. **Conclusions:** In a relatively small non-supplemented population of blood donors living in a high polluted urban area of Northern Italy, seasonal vitamin D synthesis seems to be preserved. These updated data show higher 25(OH)D levels compared to past findings. Although these data certainly warrant further validation through a national survey involving other regions of Italy and in not selected population, they appear to be in line with the SIOMMMS recommendations against indiscriminate serum 25(OH)D testing and against routine supplementation for healthy normal-weight individuals under 70 years.

## 1. Introduction

Vitamin D plays a relevant role in maintaining a healthy mineralized skeleton and in preventing rickets and osteomalacia [1]. Humans obtain vitamin D, either as vitamin D_2_ (ergocalciferol) or vitamin D_3_ (cholecalciferol), primarily through exposure to sunlight and, to a lesser extent, from dietary sources. After entering the bloodstream, vitamin D undergoes hepatic hydroxylation to 25-hydroxyvitamin D [25(OH)D], followed by a further hydroxylation step that generates 1,25-dihydroxyvitamin D [1,25(OH)_2_D], the biologically active hormone [2].

Low 25(OH)D levels (<50–60 nmol/L) have been consistently associated across different populations with an increased risk of skeletal complications, such as fractures, stress fractures and reduced bone mineral density. The effects of Vitamin D on the modulation of cytokine production, antimicrobial peptide synthesis and renin-angiotensin system balance support a biologically plausible link to other extra skeletal conditions [3]. Observational evidence suggests a possible correlation between low serum 25(OH)D concentrations and higher risk of cardiovascular disease, diabetes, cancer, autoimmune disorders, respiratory infection and all-cause mortality [3].

The measurement of serum 25(OH)D, which includes 25(OH)D2 and 25(OH)D3 forms, is used in clinical practice to assess the so-called vitamin D status and is interpreted as an expression of the body “vitamin D reserve”. In fact, the 25(OH)D form is relatively stable in serum with a half-life of 2–3 weeks, while its activated form, 1,25(OH)_2_D, has a half-life of about 15 h [4]. Vitamin D status decreases with aging, in particular after the age of 75 years. Age-related vitamin D status impairment has been ascribed to several factors, among which impaired biosynthesis due to both reduced biosynthesis capacity and lower sun exposure, but also to low dairy and fish consumption, and to increased body weight [5,6]. Population-based data from the National Health and Nutrition Examination Survey (NHANES) study in the United States of America indicate that 24% of 3.377 adults aged 40–59 years and 22% of 3.602 adults aged ≥60 years show serum 25(OH)D concentrations below 50 nmol/L; furthermore, 5.9% and 5.7% of individuals in those age groups, respectively, exhibits 25(OH)D concentrations below 25 nmol/L, with similar values observed in women and men [7]. Population-based data from European Countries (ODIN study) showed a higher prevalence of low vitamin D status in children and adults of all ages, with 40% having values below 50 nmol/L and 13% having values lower than 30 nmol/L, with similar rates in women and men [8]. In Italy, a prevalence of about 35% of adults with 25(OH)D lower than 50 nmol/L and about 13% with 25(OH)D lower than 25 nmol/L was reported [9]. In the early 2000s, Adami et al. [10] reported that among individuals aged 50–80 years the mean winter 25(OH)D serum concentrations were 38 nmol/L. Similarly, Isaia et al. [11] reported that, during the same season, mean 25(OH)D levels were approximately 25 nmol/L, and that 76% of the study population had 25(OH)D concentrations below 30 nmol/L. Among premenopausal women living in Northern Italy it was reported that 25(OH)D mean levels obtained between August and December were 69.0 ± 27.8 nmol/L, therefore highlighting the significant prevalence of young women with relatively low vitamin D levels even in a favorable season [12]. Those epidemiological observations had led to a widespread supplementation policy and to a more extensive indication to perform laboratory testing for 25(OH)D levels in the general population. In the United States of America, the prevalence of supplemental vitamin D at the dosage of 1000 IU (25 μg) or more per day increased from 0.3% in the 1999–2000 National Health and Nutrition Examination Survey (NHANES) to 18.2% in the 2013–2014 NHANES [13]. The clinical practice of testing 25(OH)D has also been subsequently increasing even if the cost-effectiveness of widespread testing has been questioned, especially considering the uncertainty about the optimal 25(OH)D levels required to prevent vitamin D-insufficiency related disease. In 2022, the Italian Society for Osteoporosis, Mineral Metabolism and Skeletal Diseases (SIOMMMS) defined, for the general healthy population and exclusively for the bone health outcome, serum 25(OH)D levels < 25 nmol/L as deficiency [14], consistently with the recommendations of other European guidelines [15,16,17].

The widespread practice of using cholecalciferol supplementation across all age groups in the general population in many countries, originally driven by the concept of a pandemic vitamin D deficiency, particularly within the Mediterranean countries, based on data published between 2000 and 2005, now makes it difficult to reassess the true current prevalence of hypovitaminosis D as compared with the estimates reported in those earlier studies [7,8,9,18]. This epidemiological picture has made it challenging to evaluate the contribution of 25(OH)D cutaneous synthesis related to solar exposure—an important physiological determinant of circulating 25(OH)D levels—and therefore to investigate its seasonal variability, also in light of the evidence regarding the impact of air pollution, especially the presence, in the atmosphere, of high concentrations of particulate matter (PM_10_, PM_2.5_) and nitrogen dioxide (NO_2_). Elevated concentrations of fine particulate matter were shown to act as a filter for ultraviolet radiation, significantly reducing the potential for cutaneous synthesis of 25(OH)D [19,20,21].

For those reasons, it is of particular interest to update the knowledge on vitamin D status in an urban population living in Northern Italy and that is characterized for not receiving vitamin D supplementation. The data described here were obtained from a population living in Verona, a city located in the Region of Veneto, situated in the Po Valley, an area of Northern Italy well-recognized for the weak intensity of solar irradiation (3600–4700 MJ/m^2^, 60–80% lower than those in Southern Italy) [22] and, according to the European Environment Agency, one of the most polluted regions in Europe over the least 20 years, with persistently high concentrations of PM_10_, PM_2.5_ and NO_2_ throughout all seasons [23,24].

It is reasonable, therefore, to hypothesize that the scenario emerging from the present study may represent the nadir of vitamin D status in Italy across the different seasons. On this basis, it is also of interest to verify, in a real-life setting, the applicability of the recommendations from the recent national guidelines of the SIOMMMS [14].

## 2. Materials and Methods

### 2.1. Ethical Standards

The study was approved by the Verona University Hospital Ethical Committee (VIT-9612CROSS, Prog.779CESC). Eligible subjects were informed about the objectives and procedures of the study. All participants provided written informed consent before enrolment. The procedures used were in accordance with the ethical standards of the responsible institutional or regional committee on human experimentation, or with the Helsinki Declaration of 1975, as revised in 1983. Data used for the study were completely anonymized.

### 2.2. Study Design and Participants

Study design has already been reported elsewhere [25,26]. This cross-sectional study had the original intent of evaluating levels of folate, being one of the major determinants for the role of dietary nutrients for major disease risk. Briefly, from April 2016 to May 2018, 551 healthy blood donors, consecutively attending the Transfusion Medicine Unit of the Verona University Hospital (Italy) were considered for inclusion in this cross-sectional study, 538 of whom (97.6%) agreed to participate. The only exclusion criteria were vitamins supplementation during the two months before the blood sample withdrawal. Two subjects were excluded due to ongoing vitamin D supplementation, and two patients were excluded due to pre-analytical drawbacks, for a final population of 534 patients (268 men and 266 women, of whom 154 of childbearing age, between 18 and 44 years) included in the study. After giving written informed consent, each participant was interviewed about her/his general characteristics, medical history, and current therapy. Lifestyle and education, as recognized parameters of lifestyle and dietary habits, including alcohol and smoking habits, were also recorded. In this study we retrospectively analyzed the vitamin D values collected in the original cross-sectional study.

Vitamin D levels were analyzed according to the seasons, defined on the basis of the conventional equinoxes and solstices at our latitude, and according to each month of the year, i.e., from January to December.

### 2.3. Laboratory Parameters

Venous whole blood samples were collected after overnight fasting into Vacutainer^®^ tubes (BD-Becton, Dickinson and Company; Franklin Lakes, NJ, USA) without anticoagulant to obtain serum. After centrifugation at 1500× *g* for 10 min at room temperature, serum was separated, stored in aliquots and kept frozen at −70 °C until measurement.

Serum concentrations of 25-hydroxyvitamin D [25(OH)D] were measured using the LIAISON Analyzer (DiaSorin S.p.A., Saluggia, Italy) with the vitamin D TOTAL Assay (DiaSorin S.p.A., Saluggia, Italy). This assay is a direct competitive chemiluminescent immunoassay (CLIA) designed for the quantitative determination of total 25(OH)D, including both 25(OH)D2 and 25(OH)D3, in human serum. The different values for serum 25(OH)D concentrations allowed to define the 25(OH)D status as follows: deficiency if <25 nmol/L. insufficiency when ranging 25–49 nmol/L, optimal if ≥50 nmol/L; values >250 nmol/L were considered as 25(OH)D excess [27]. The samples were analyzed according to the manufacturer’s instructions and the internal laboratory guidelines.

### 2.4. Statistical Analysis

Data were collected in a specific database after a review for completeness, consistency, and plausibility. This study included 534 subjects. The original sample size was computed considering, as the primary objective of the study, the frequency of adequate plasma folate concentrations (>15 nmol/L) [25,26]. Considering the endpoint of the present analysis (i.e., the prevalence of adequate vitamin D status) based on a post hoc computation, we were able to determine estimates of adequate serum vitamin D concentrations with a narrow 95% confidence interval (CI). Continuous variables were reported by mean values and standard error. Categorical variables were presented by calculating absolute frequency and percentage. The 95% CI of the mean and proportion were provided to assess the precision of estimates. All continuous variables were compared between subgroups using the analysis of variance (ANOVA) or the Kruskal–Wallis test when appropriate. The analysis of covariance (ANCOVA) was used to adjust the values for age, gender and BMI. To discriminate among the means, Fisher’s least significant difference (LSD) procedure was used. Odds Ratios (ORs) for inadequate status of vitamin D according to sociodemographic and general characteristics were computed. Chi-square tests were used for categorical data. Associations between continuous variables were examined using Pearson correlation coefficients. Differences were considered significant at *p* < 0.05. All statistical procedures were carried out using a computer program (Statgraphic Centurion v19, by Statgraphics Technologies, Inc., The Plains, VA, USA).

## 3. Results

The population of this study was selected from the participants of the study from Bortolus R. et al. [25]. The demographic and anthropometric characteristics of this study population are reported in Table 1. The study population had a mean age of 42 years (95% CI: 40.9–43.0). Females represented 49.8% of the study population (266/534) and were younger than males (mean age 38.0 years; 95% CI: 37.0–39.0 vs. 46.0 years; 95% CI: 45.0–46.9, respectively; *p* = 0.001). Females also had a significantly lower BMI than males (BMI 22.6; 95% CI: 22.3–22.9 vs. 25.2; 95% CI: 25.0–25.5, respectively; *p* = 0.001).

The mean 25(OH)D serum concentrations from January to December was 57.0 nmol/L (95% CI: 55.0–65.8). No significant differences were observed for gender (female: 57.5 nmol/L, 95% CI: 55.5–59.5 vs. male: 56.7 nmol/L, 95% CI: 54.5–58.6; *p* = 0.622). No significant changes were observed adjusting 25(OH)D levels for age and BMI (Table 1).

Analysis of the distribution of 25(OH)D levels in the overall population showed that 7.1% (38/534) of subjects had levels <25 nmol/L, 31.1% had levels between 25 and 49 nmol/L (166/534) and 61.8% (330/534) had 25(OH)D ≥ 50 nmol/L, i.e., in the optimal range, with no differences between sexes (Chi-square 0.399; Table 1). Stratifying the 25(OH)D levels by seasons we found that there was a clear effect of seasonality with a nadir in winter (48.3 nmol/L, 95% CI 45.5–51.0), with values significantly lower than those found in summer and autumn, and a peak in autumn (70 nmol/L; 95% CI: 66.3–73.5), even after adjustment for age and BMI. This effect was also evident after stratifying by sex, with a non-significant trend toward lower levels in winter, and slightly higher levels in autumn for males vs. females (Table 1).

No correlation was found between age and 25(OH)D levels in the overall population (R^2^ = 0.04; *p* = 0.603), nor when the analysis was stratified by sex.

When comparing the annual 25(OH)D mean concentrations across age quartiles, no significant difference was observed between the youngest quartile (138 subjects aged <35 years; mean 57.5 nmol/L; 95% CI: 54.8–60.5) and the oldest quartile (141 subjects aged >51 years; mean 57.9 nmol/L; 95% CI: 55.2–60.5; *p* = 0.84 between groups). Similarly, when 25(OH)D levels were compared across age quartiles at nadir reached in February, no significant differences were detected with mean values of 39.5 nmol/L (95% CI: 30.5–48.3) in the <35 years quartile and 43.8 nmol/L (95% CI: 36.0–51.5) in the >51 years quartile (*p* = 0.392).

**Figure 1:**
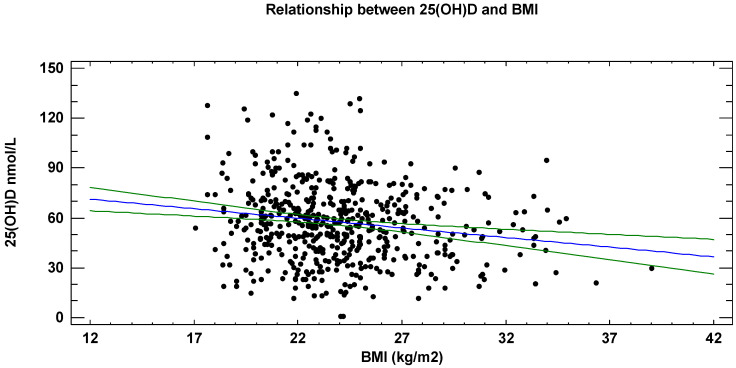
Relationship between serum 25(OH)D (nmol/L) and BMI (kg/m^2^) (*p* = 0.001). The central blue line is the best fit regression line, the two green lines represent the confidence bands.

BMI showed a negative correlation with 25(OH)D levels in the overall population (R^2^ = 3%; *p* = 0.001) (Figure 1), with a similar correlation observed in males and females.

A more detailed analysis of distribution of 25(OH)D levels by months in the overall population, after adjustment for age and BMI, across the year showed the nadir in February (43.7 nmol/L; 95% CI: 38.5–48.7) and the peak values between August and September (83.0 nmol/L; 95% CI: 72.3–93.3) (*p* = 0.0014). In February, approximately 41.5% of the overall population had levels ≥ 50 nmol/L, and only 27.7% of subjects had 25(OH)D levels < 25 nmol/L, while 30.8% had values between 25 and 49 nmol/L (Table 2). There were no significant differences in absolute values of 25(OH)D by sex across the year but slight differences in the timing of nadir.

**Figure 2:**
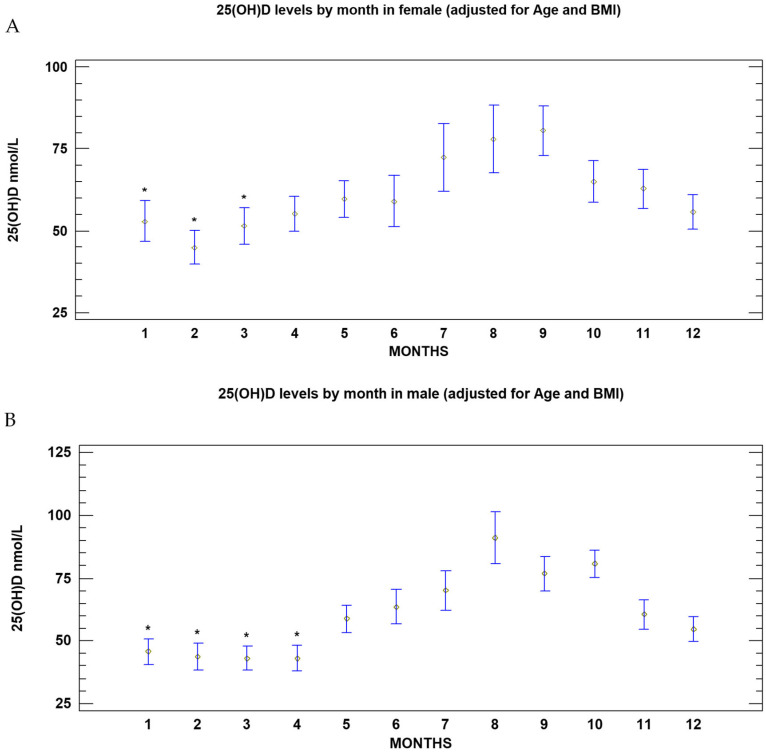
(**A**): Serum 25(OH)D concentrations (nmol/L) by month in the female subgroup (mean and 95% CI) adjusted for age and BMI. * *p* = 0.002 vs. months from July to October. (**B**): Serum 25(OH)D concentrations (nmol/L) by month in the male subgroup (mean and 95% CI) adjusted for age and BMI. * *p* = 0.001 vs. months from May to December.

In females, the nadir of 25(OH)D, adjusted for age and BMI, occurred in February (44.3 nmol/L; 95% CI: 37.0–51.5). Mean 25(OH)D levels in January, February and March were significantly lower than those observed in July, August, September, and October (*p* = 0.002). The values of vitamin D peaked in September (82.0 nmol/L; 95% CI: 71.0–93.0; Figure 2A). At the nadir 40.6% of female had 25(OH)D ≥ 50 nmol/L, 37.5% between 25 and 49 nmol/L, and only 21.9% of females had levels < 25 nmol/L (Table 2).

In males, the nadir of 25(OH)D concentrations adjusted for age and BMI, occurred in March (42.3 nmol/L; 95% CI: 35.3–48.5). Mean 25(OH)D levels in January, February, March and April resulted significantly lower than those observed from May to December (*p* = 0.001); mean 25(OH)D concentrations in May also resulted significantly lower than those observed in August (*p* = 0.001). The 25(OH)D peaked in August (90.3 nmol/L; 95% CI: 75.8–104.7; Figure 2B). Nevertheless, in males during the period of nadir, 42.4% of subjects had 25(OH)D levels ≥ 50 nmol/L, 24% between 25 and 49 nmol/L, and finally 33.3% of subjects had levels < 25 nmol/L (Table 2).

**Figure 3:**
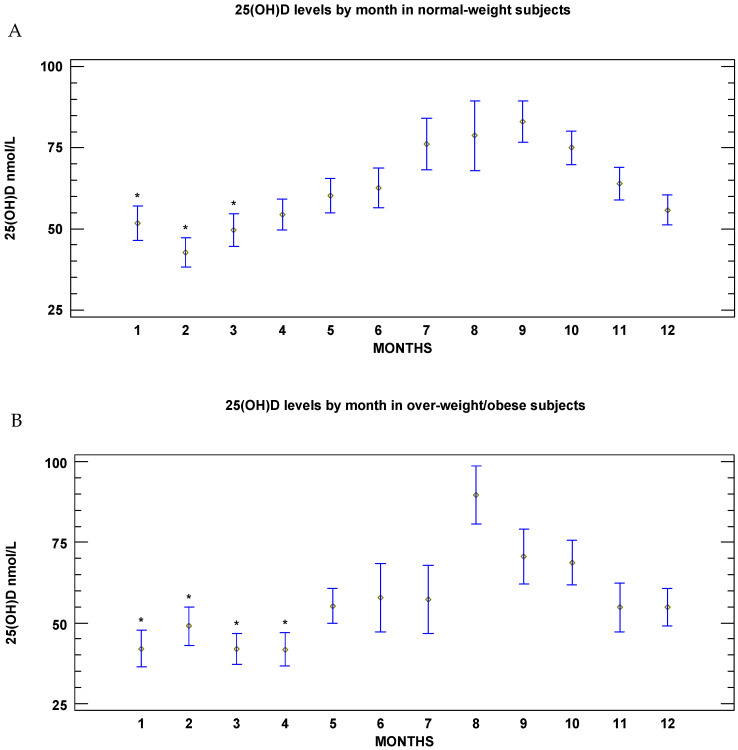
(**A**): Serum 25(OH)D concentrations (nmol/L) by month in the normal weight subgroup (mean and 95% CI). * *p* = 0.001 in January vs. months from July to November, in February vs. months from April to December, in March vs. months from May to December. (**B**): Serum 25(OH)D concentrations (nmol/L) by month in the overweight/obese subgroup (mean and 95% CI). * *p* = 0.001 in January, March and April vs. months from May to December, in February vs. months from August to October.

The monthly changes in vitamin D across the year, when stratifying by BMI level (normal weight and overweight/obese), were quite different between normal-weight subjects and overweight/obese subjects. The demographic data are reported in Table 3. Among normal weight subjects, the nadir occurred in February (mean 25(OH)D 42.5 nmol/L; 95% CI: 40.0–44.8), and the peak value was observed in September (78.0 nmol/L; 95% CI: 74.0–91.8). From March to January, the mean 25(OH)D levels were ≥50 nmol/L (*p* = 0.001; Figure 3A).

In the overweight/obese subgroup, the mean value of BMI was 28.0 (95% CI: 27.7–28.2) kg/m^2^. The overweight subjects were 79.3% (130/164), and the obese were 20.6% (34/164). Overweight/obese subjects were significantly older than their normal weight counterparts (44.1 years; 95% CI: 42.8–45.4 vs. 41.0 years 95% CI: 40.2–41.9; *p* = 0.002) and showed significantly lower serum 25(OH)D concentrations (52.0 nmol/L 95% CI: 49.5–54.5 vs. 59.3 nmol/L 95% CI: 57.7–61.0; *p* = 0.002). In overweight/obese subjects, the mean 25(OH)D levels remained <50 nmol/L from January to April in almost all individuals (36.3 nmol/L; 95% CI: 33.8–39.5 and 38.3 nmol/L 95% CI: 34.8–41.3, respectively; *p* = 0.001). Approximately 45% of these subjects still had levels < 50 nmol/L from May to July (mean values ranging, respectively, from 54.5 nmol/L 95%; CI: 46.8–62.3 to 56.0 nmol/L; 95% CI: 45.2–66.5). The highest 25(OH)D concentrations were observed in August (88.5 nmol/L; 95% CI: 75.8–101.3; Figure 3B). In the overweight/obese subgroup the prevalence of subjects with 25(OH)D deficiency (10.3%, 17/164) and insufficiency (36.6%, 60/164) was slightly but not significantly higher compared with the normal weight subgroup (deficiency 7.3% (27/370) and insufficiency 29.2% (108/370) (Chi-square 0.052). When evaluating 25(OH)D levels at nadir, a non-significant reduced prevalence of patients with optimal values was observed in the overweight/obese subgroup compared with normal weight subjects (42.5% (17/40) vs. 62.0% (44/71), respectively; Chi-square 0.08); Table 3.

When exploring the population data for lifestyle, eating habits and socio-cultural aspects, no significant risk factors for low vitamin D (considered for 25(OH)D < 50 nmol/L) were observed s (Table 4).

## 4. Discussion

Our data provide the first evidence in more than 20 years [10,11,28] on serum vitamin D concentrations and their seasonal variation in a large population of blood donors from Northern Italy not receiving vitamin D supplementation, thus reflecting endogenous synthesis, which is known to depend almost entirely on sun exposure. This is particularly relevant in Italy, where fortification of foods with vitamin D is not widespread and vitamin D-fortified foods are rarely consumed by the general population due to the high cost. Our findings confirm the persistence of the “physiological” seasonal rhythm in serum 25(OH)D synthesis in healthy adults of both sexes, at least in the age group analyzed (range 18–65 years). While this finding may seem a rather marginal information, the interest in updating the epidemiological information about 25(OH)D levels and exploring the persistence of physiological synthesis of vitamin D in Northern Italy arises from the data supporting an impact of air pollutants on the penetration of solar ultraviolet B (UVB) to the Earth’s surface, therefore linking the occurrence of vitamin D deficiency to air pollution [21]. Secondly, the recently published recommendations from SIOMMMS, consistently with the 2024 Endocrine Society guidelines, indicate a value of 25(OH)D equal to or greater than 50 nmol/L to be considered optimal in the general population, deeming unnecessary a universal supplementation with cholecalciferol in the healthy general population [14,29]. Therefore, it was of interest to document how consistent the recommendations from the Italian and International guidelines are with vitamin D status in the Italian healthy adult population.

The samples for the analysis of serum 25(OH)D were collected, among the blood donors recruited, between April 2016 and May 2018, thus covering all four seasons. People recruited lived in the urban area of Verona, located in North-Eastern Italy (45°26′19″ N, 10°59′34″ E), about 59 m above the sea level. The city covers an area of 198.92 km^2^ and is densely populated (1283.42 inhabitants/km^2^). Noteworthy, Verona is located in the Po Valley, a geographical area burdened by some of the highest concentrations in the European continent of fine particular air pollution across all seasons [23,24].

In the autumn/winter season, pollution is mostly represented by PM_10_ and PM_2.5_ and in spring/summer by Ozone (O_3_), benzo(a)pyrene and NOx. For all these pollutants, there is evidence for blocking UVB on the Earth surface and subsequently preventing the cutaneous synthesis of vitamin D [19,20,21]. Surprisingly, in our study, atmospheric pollution did not appear to meaningfully compromise the endogenous vitamin D synthesis throughout the year, and the 25(OH)D seasonal variations are well preserved and perfectly comply with what is expected from the literature [28]. In our study, serum 25(OH)D levels were lower during winter, reaching a nadir of approximately 45 nmol/L in February–March, and progressively increased during spring and summer, peaking between August and September at about 82.5 nmol/L. Previous studies conducted in areas of Italy at different latitudes have similarly documented a clear seasonal pattern in serum 25(OH)D concentrations, with lower values detected in winter and higher values during summer [30,31]. A comparable seasonal trend has also been reported in European countries at different latitudes, including Finland, the United Kingdom, Germany, Spain and Turkey [28,32,33].

When compared with epidemiological studies on hypovitaminosis D conducted in Italy in the early 2000s [11,34,35], serum 25(OH)D concentrations appear to be significantly higher in the present cohort, with a marked reduction in the overall prevalence of hypovitaminosis D. This gap appears particularly evident when considering the prevalence of vitamin D deficiency (25(OH)D < 25 nmol/L) and insufficiency (25(OH)D < 50 nmol/L). Manios Y. et al. found in Italy a prevalence of about 35% of adults with 25(OH)D lower than 50 nmol/L, with about 13% of those with 25(OH)D lower than 25 nmol/L [9]. It should be highlighted that those data were collected in the Central/Southern area of Italy and represented a mean yearly value [9]. In the early 2000s, Adami S. et al. [10] reported that, among individuals aged 50–80 years, the mean 25(OH)D concentrations in winter was 38 nmol/L. Isaia et al. [11] observed, in the same season, mean 25(OH)D levels of about 25 nmol/L, with a prevalence of 25(OH)D < 30 nmol/L of 76% in the population studied. In Northern Italy, among premenopausal women (ranging 28–44 years of age) between September and December 2006, mean 25(OH)D levels of 69.0 ± 27.8 nmol/L were documented, therefore showing a significant prevalence of young women with relatively low vitamin D levels in a favorable season for UVB exposure [12]. These data induced the promotion, in Italy, of a widespread supplementation with cholecalciferol in the general population [18]. In the present study, we found that about 60% of females and males have optimal (≥50 nmol/L) 25(OH)D concentrations in all the seasons, only 7.1% of the subjects had 25(OH)D levels lower than 25 nmol/L, and 31.1% had 25(OH)D levels between 25 and 49 nmol/L. In particular, females in our study have the nadir levels of vitamin D in February. Anyway, in the worst month of the year for 25(OH)D skin synthesis, 40.6% of the females had 25(OH)D ≥ 50 nmol/L. In males, the nadir occurred in March with 42.4% of subjects having vitamin D levels ≥ 50 nmol/L. Because of the well-known negative impact of adiposity on 25(OH)D levels, when overweight and obese subjects were excluded from the analysis, the nadir occurred in February (mean 25(OH)D 42.5 nmol/L (95% CI: 40.0–44.8), whereas from March to January almost all subjects had 25(OH)D levels ≥ 50 nmol/L [36,37,38,39,40]. It is possible that factors such as higher scholarity, lifestyle, social behavioral changes over the past decades may have played a role. In our study, none of these factors was associated with vitamin D insufficiency, unlike what has been reported in previous studies [10,11,12].

In the literature a “Healthy Donors Effect” bias has been reported, with better health outcomes observed in the blood donor population [41]. Previous studies highlighted how blood donors, compared with the general population, tend to have better lifestyle habits, lower prevalence of smoking and higher self-reported hours of physical activity [42,43]. Consistently, the majority of the patients in our population did not smoke and practiced >2 h/week of physical activity. Smoking seems to negatively influence serum 25(OH)D concentrations in both supplemented and non-supplemented subjects [44], but in our study we did not observe a correlation between smoking habits and serum 25(OH)D concentrations. Evidence regarding the impact of physical exercise on vitamin D levels is controversial, and the possible positive effect seems to be influenced by the hours spent outdoor [45,46]. Our findings did not show a significant association between weekly hours of physical activity and serum 25(OH)D levels. It is possible that other factors not evaluated in the present study may have played a role.

**Figure 4:**
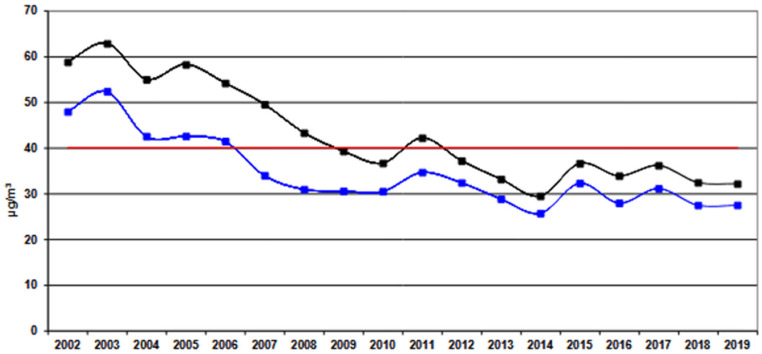
Mean PM10 (ug/m^3^) concentrations in the air per year as revealed by industrial (black line) and background (blue line) monitoring stations of the Region of Veneto. The red line indicate the threshold values defined by Law (data from ARPAV as per license CC BY 3.0) [47].

A suggestive explanation for the apparent improvement in vitamin D status among individuals living in the same geographic area where previous studies were conducted approximately 10 years later may be the progressive and substantial reduction in environmental pollution that started around 2014 in the region, separating the earlier studies from the present investigation [10,11]. The 2020 local environmental reports highlighted a 46% reduction in PM_10_ concentration and 38% reduction in NO_2_ levels between 2005 and 2019 in Verona, with a similar trend also for PM_2.5_. Noteworthy, mean levels of these pollutants dropped below the recommended thresholds, giving a possible explanation for the absent influence of pollution on 25(OH)D concentrations observed in the present study (data from ARPAV as per license CC BY 3.0, Figure 4) [47].

Finally, it should be emphasized that the study population had been carefully selected from individuals not taking nutritional supplements or any form of vitamin D supplementation.

The findings of our study seem to support the recommendations expressed by SIOMMMS and the guidelines of the Endocrine Society, which defined as optimal a level of vitamin D as equal or above 50 nmol/L and advises against empirical vitamin D supplementation in healthy individuals under 70 years of age [14,29]. As described above, when overweight and obese subjects were excluded, the nadir of 25(OH)D levels in the normal-weight population occurred in February, and the 25(OH)levels were very low. Furthermore, this was the only month in which the majority of subjects had 25(OH)D levels < 50 nmol/L. From March to January, almost all subjects had 25(OH)D levels ≥ 50 nmol/L. It is unlikely that only one month with 25(OH)D levels < 50 nmol/L may significantly affect bone and mineral metabolism or other potential extra skeletal health outcomes. Our data, therefore, support the choice not to empirically supplement healthy, normal-weight individuals aged ≤70 years and not to perform universal screening for serum 25(OH)D levels [14,29].

We also did not find any association of vitamin D levels with age in either males or females.

When comparing the annual mean 25(OH)D concentrations across age quartiles, no significant difference was observed between the youngest and the oldest age quartiles (<35 years vs. >51 years), and no differences were observed in the nadir values in February. There is evidence that the synthesis of vitamin D declines progressively with aging, and previous studies reported an inverse correlation between age and serum 25(OH)D concentrations, starting above 60 years of age in female and above 70 years of age in male [5]. Our results may be explained by the limited number of participants aged between 60 and 65 years.

Furthermore, as it is well known, vitamin D levels are negatively influenced by BMI, as we also found that the seasonal pattern of 25(OH)D levels is also affected by BMI [36,37,38,39,40]. When stratifying the population according to BMI, in overweight/obese subjects, 25(OH)D levels remained <50 nmol/L from January to April in almost all subjects and approximately 45% of those subjects maintained levels < 50 nmol/L between May and July without gender differences. It is noteworthy that, in the overweight/obese group, overweight subjects are more prevalent than obese subjects (130/164 vs. 34/164, respectively) and the mean BMI was in the range of overweight BMI classification. In other words, it is likely that in a cohort of exclusively obese subjects, the seasonal vitamin D changes may be worse.

The need for supplementation policy and the issues of obtaining adequate serum levels in overweight/obese people are widely debated in the literature [36,39,48,49]. Our data seem to support the recommendations of the Italian guidelines, which include obesity among the conditions for high risk of hypovitaminosis D and, therefore requiring empirical supplementation [14].

Weaknesses of the present study include the retrospective analysis of a cross-sectional study. Another possible limitation is the age range (18–65 years), which prevents us from exploring the ability to synthesize vitamin D in elderly subjects. On the other end this limitation excludes bias due to low mobility, comorbidity and polypharmacy. Another limitation could be the population selected among blood donors who are likely healthier and have a better lifestyle than the general population, that may represent a possible bias that could determine higher levels of vitamin D compared with the general population. Finally, we did not obtain data regarding the direct impact of environmental pollution on vitamin D synthesis, which then remains a speculative aspect.

A strength of this study is instead represented by the careful selection of healthy, free-living subjects who were not taking any vitamin D supplements, a rather uncommon condition that hampers epidemiological studies on vitamin D synthesis. It is also to be remarked that the relatively homogeneous distribution of the sampling of serum 25(OH)D across the year allowed the creation of a curve showing the monthly mean levels of 25(OH)D, a kind of data quite rare in the recent literature. Another strength is that the present study was carried out in a region geographically and ecologically disadvantaged, both for a 60–80% lower solar radiation compared to Southern regions of Italy [22], and for the higher levels of pollution [24], both of which factors that significantly influence the synthesis of vitamin D. It seems reasonable, therefore, to assume that other regions of Italy may have a better vitamin D status. Such hypothesis does not exclude but rather support the need for a national survey to confirm our findings. This finding is indeed of importance in terms of public health intervention policies related to vitamin D.

## 5. Conclusions

In conclusion, our data demonstrate that seasonal variations in synthesis of vitamin D and adequate levels of vitamin D across the year are preserved in a high-polluted urban area. In this selected population of blood donors, in normal weight female and male the prevalence of vitamin D insufficiency (25(OH)D < 50 nmol/L) is very low and limited to one-two months of the year. Overweight and obesity significantly preclude the achievement of adequate serum 25(OH)D concentrations with the sole cutaneous synthesis for many months during the year. Although these data certainly warrant further validation through a national survey involving other regions of Italy, they appear to support the SIOMMMS recommendations against indiscriminate serum 25(OH)D testing, to avoid empirical supplementation in healthy normal-weight individuals under 70 years of age while providing evidence for supplementation for overweight/obese individuals.

## Figures and Tables

**Table 1:** Demographic characteristic of subjects and 25(OH)D levels across the seasons (data are expressed as mean and CI 95%).

	All	Female	Male	ANOVA *p* Value Between Genders
*n*	534	266	268	
Age	42.0 (40.9–43.0)	38.0 (37.0–39.0)	46.0 (45.0–46.9)	0.001
BMI	23.9 (23.6–24.2)	22.6 (22.3–22.9)	25.2 (25.0–25.5)	0.001
25(OH)D nmol/L	57.0 (55.0–65.8)	57.5 (55.5–59.5)	56.7 (54.5–58.6)	0.622
25(OH)D nmol/L ^#^	56.8 (54.8–60.3)	56.0 (53.0–58.8)	58.3 (55.3–61.0)	0.456
Spring ^#^	54.5 (50.8–58.3)	56.8 (51.5–62.3) *	52.0 (46.5–58.0) ^a^	0.446
Summer ^#^	68.8 (63.5–74.0)	66.8 (58.5–73.0) **	70.3 (63.5–77.0) ^b^	0.919
Autumn ^#^	70.0 (66.3–73.5)	67.5 (62.0–73.0) **	72.3 (67.3–77.3) ^b^	0.190
Winter ^#^	48.3 (45.5–51.0)	50.3 (46.5–54.3)	46.3 (42.5–49.8)	0.284
**25(OH)D status ^**				**Chi-square**
Deficiency (<25 nmol/L)	7.1% (38)	6.8% (18)	9.7% (26)	
Insufficiency (25–49 nmol/L)	31.1% (166)	33.1% (88)	29.9% (80)	0.399
Optimal (≥50 nmol/L)	61.8% (330)	60.1% (160)	60.4% (162)	

^#^ 25(OH)D levels (nmol/L) adjusted for age and BMI; ^ According to 2022 SIOMMMS guidelines [14]. Chi-squared 0.399, Comparison of prevalence of different 25(OH)D levels between female and male; * *p* = 0.0001, 25(OH)D in spring vs. summer and autumn.; ** *p* = 0.001, 25(OH)D in summer and autumn vs. winter; ^(a)^ *p* = 0.002, 25(OH)D in spring vs. summer and autumn; ^(b)^ *p* = 0.001, 25(OH)D in summer and autumn vs. winter.

**Table 2:** Subjects with vitamin D deficiency (<25 nmol/L), insufficiency (25–49 nmol/L) and optimal levels (≥50 nmol/L) according to SIOMMMS criteria [14] at nadir (February/March).

25(OH)D Levels at NADIR	All (65)	FEMALE (32)	MALE (33)
Deficiency % (*n*)	27.7 (18)	21.9 (7)	33.3 (11)
Insufficiency % (*n*)	30.8 (20)	37.5 (12)	24.3 (8)
Optimal % (*n*)	41.5 (27)	40.6 (13)	42.4 (14)

Chi-squared 0.908, Comparison of the prevalence of different 25(OH)D levels at nadir between females and males.

**Table 3:** Demographic characteristics and 25(OH)D levels by SIOMMMS guidelines criteria [14] in the participants of the study stratified by BMI (normal weight or overweight/obese).

	Normal Weight (370)	Overweight/Obese (164)	*p* Value
F/M	213/157, F 57.6%	53/111, F 32.3%	
Age	41.0 (40.2–41.9)	44.1 (42.8–45.4)	0.002
BMI kg/m^2^	22.2 (22.0–22.3)	28.0 (27.7–28.2)	0.001
25(OH)D nmol/L	59.3 (57.7–61.0)	52.0 (49.5–54.5)	0.002
Deficiency % (*n*)	7.3 (27)	10.3 (17) ^a^	
Insufficiency % (*n*)	29.2 (108)	36.6 (60) ^a^	
Optimal % (*n*)	63.5 (235)	53.0 (87) ^a^	
Nadir 25(OH)D (nmol/L)	42.5 (40.0–44.8)	41.3 (35.0–47.5)	0.337
Deficiency (% at nadir)	4.2 (3/71)	10.0 (4/40) ^b^	
Insufficiency (% at nadir)	33.8 (24/71)	47.5 (19/40) ^b^	
Optimal (% at nadir)	62.0 (44/71)	42.5 (17/40) ^b^	

Data expressed as mean (95% CI); F: female, M: male, ^a^ Chi-squared 0.052, comparison of prevalence of different 25(OH)D levels in normal weight vs. overweight/obese. ^b^ Chi-squared 0.08, comparison of prevalence of different 25(OH)D levels at nadir between normal weight and overweight/obese.

**Table 4:** Educational attainment and lifestyle characteristics in the overall cohort and in subjects with vitamin D insufficiency (25(OH)D < 50 nmol/L).

		25(OH)D <50 nmol/L	
	*N* TOT	*N* (%)	OR (95% CI) *
Education (y)			
≤8	82	37 (18.1)	1 +
9–15	325	120 (58.8)	0.9 (0.55–1.62)
≥16	127	47 (23.1)	0.9 (0.50–1.81)
Current smoker			
No	459	173 (84.8)	1 +
Yes	75	31 (15.2)	1.2 (0.69–1.96)
Alcohol drinking			
No	167	60 (29.4)	1 +
Yes	370	144 (70.6)	0.7 (0.44–1.11)
Fruit and Vegetable			
1 portion/day	57	28 (13.7)	1 +
2 portions/day	396	154 (75.5)	1.0 (0.68–1.62)
3 portions/day	81	22 (10.8)	0.8 (0.44–1.31)
Physical activity			
<2 h/week	215	101 (49.5)	1 +
2–5 h/week	213	67 (32.8)	0.6 (0.39–0.91)
>5 h/week	106	36 (17.7)	0.6 (0.35–1.00)

OR: odds ratio * multivariate estimates including in turn term education (years of studies), smoking, alcohol drinking, fruits and vegetables intake, physical activity. + Reference category.

## Data Availability

The original contributions presented in this study are included in the article. Further inquiries can be directed to the corresponding author. References are provided for all sources (data regarding pollution in the area of Verona [47]; European data on pollution [24]).

## Abbreviations

The following abbreviations are used in this manuscript:

25(OH)D	25-hydroxyvitamin D
BMI	Body Mass Index
CI	Confidence Interval
EDTA	Ethylenediaminetetraacetic Acid
NHANES	National Health and Nutrition Examination Survey
OR	Odds Ratio
SIOMMMS	Italian Society for Osteoporosis, Mineral Metabolism and Skeletal Diseases
